# Supplementary material for: KSMoFinder—knowledge graph embedding of proteins and motifs for predicting kinases of human phosphosites
Source: Bioinform Adv. 2025 Nov 11;5(1):vbaf289. doi: 10.1093/bioadv/vbaf289 (PMC12664573; doi:10.1093/bioadv/vbaf289)
Supplement: vbaf289_Supplementary_Data [file vbaf289_supplementary_data.zip › Supplementary_File_2.docx]

Table 1: Results of including additional features (kinase domain sequences and protein structures) via embeddings from other pre-trained models.

| **Test No.** | **Test Description** | **Features & Embedding sources** | **Testing dataset 1** | | **Testing dataset 2** | |
| --- | --- | --- | --- | --- | --- | --- |
|  |  |  | **ROC-AUC** | **PR-AUC** | **ROC-AUC** | **PR-AUC** |
| 1 | With KSMoFinder-KGE only | Protein biological associations, 9-mer motif (**KSMoFinder-KGE**) | **0.906+0.004** | **0.906+0.006** | **0.93+0.003** | **0.801+0.009** |
| 2 | Inclusion of ProstT5-based protein structure information | Protein biological associations, 9-mer motif (**KSMoFinder-KGE**)  Protein structure (ProstT5) | **0.899+0.006** | **0.888+0.008** | **0.923+0.004** | **0.782+0.004** |
| 3 | Inclusion of Phosformer-based kinase domain sequence and motif sequence information | Protein biological associations, 9-mer motif (**KSMoFinder-KGE**) Kinase domain sequence, 15-mer motif (Phosformer) | **0.898+0.006** | **0.893+0.006** | **0.925+0.003** | **0.785+0.006** |
| 4 | Inclusion of ProstT5 (structure) and Phosformer (kinase domain sequence and motif sequence) information | Protein biological associations, 9-mer motif (**KSMoFinder-KGE**) Kinase domain sequence, 15-mer motif (Phosformer)  Protein structure (ProstT5) | **0.893+0.006** | **0.886+0.008** | **0.92+0.003** | **0.777+0.009** |
| 5 | Effect of dropping KSMoFinder-KGE | Kinase domain sequence, 15-mer motif (Phosformer)  Protein structure (ProstT5) | 0.724+0.004 | 0.652+0.004 | 0.802+0.004 | 0.422+0.004 |
| 6 | Effect of dropping proteins’ biological information contributed via KSMoFinder-KGE | 9-mer motif (KSMoFinder-KGE)  Protein structure (ProstT5) | 0.831+0.005 | 0.794+0.01 | 0.876+0.004 | 0.628+0.01 |


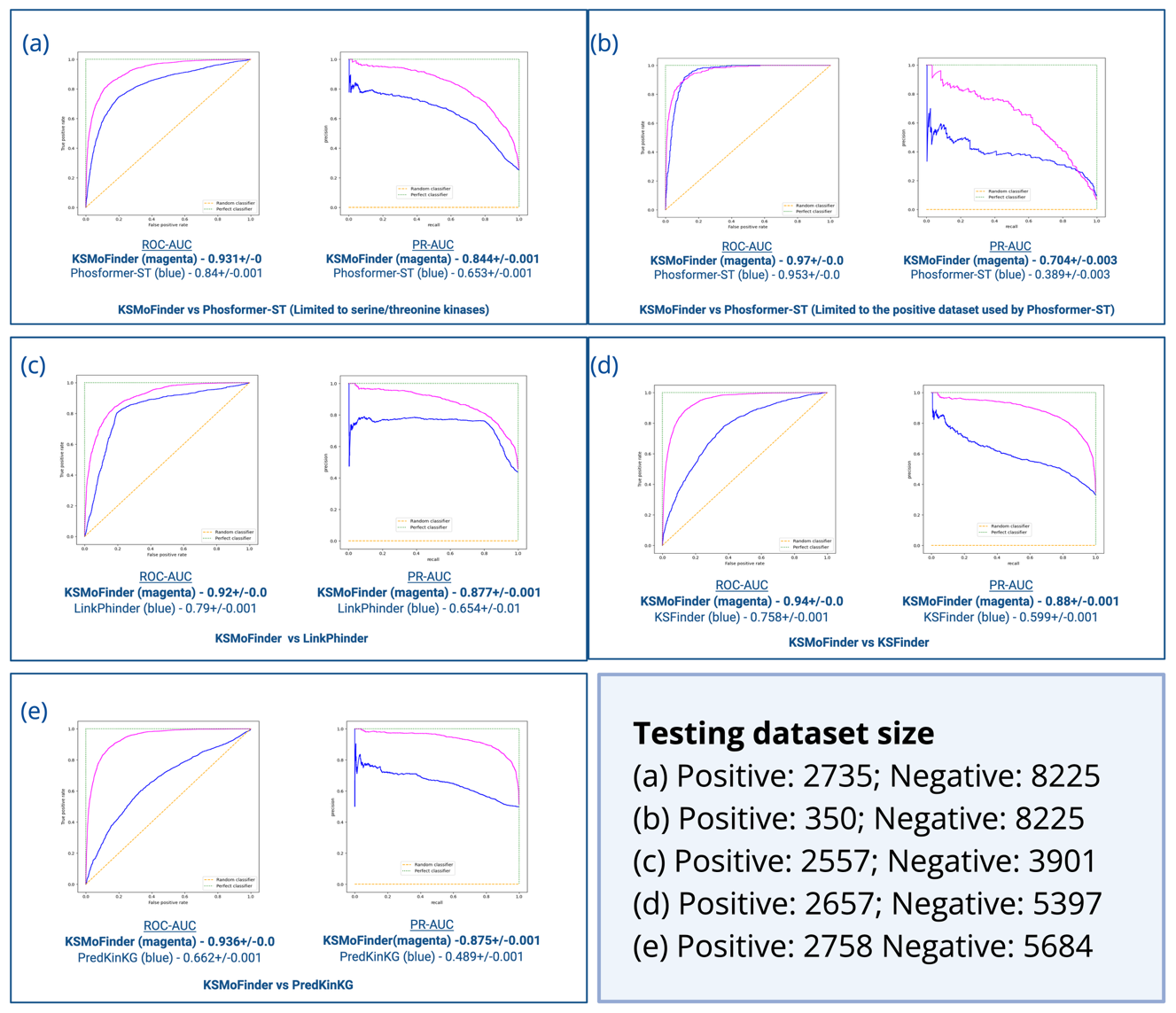


Figure 1. Pairwise comparative evaluation results of KSMoFinder with other models using Testing Dataset 2. Score are reported in ROC-AUC and PR-AUC. (a) KSMoFinder vs Phosformer-ST using the positive dataset from iPTMnet and PSP, (b) KSMoFinder vs Phosformer-ST using the subset containing positive samples reported by Johnson et. al., [4], (c) KSMoFinder vs LinkPhinder, (d) KSMoFinder vs KSFinder, (e) KSMoFinder vs PredKinKG. Created in BioRender. Anandakrishnan, M. (2025) https://BioRender.com/s8t76e0

Table 2. Comparative evaluation results of KSMoFinder KGE with other protein-based models on testing datasets with and without easy test scenarios.

| **Model** | **Without easy test scenarios** | | | | **With easy test scenarios** | | | |
| --- | --- | --- | --- | --- | --- | --- | --- | --- |
|  | **Testing dataset 1** (Positive: 2444;  Negative: 2444) | | **Testing dataset 2** (Positive: 2444;  Negative: 7240) | | **Testing dataset 1** (Positive: 3025;  Negative: 3025) | | **Testing dataset 2** (Positive: 3025;  Negative: 8489) | |
|  | **ROC-AUC** | **PR-AUC** | **ROC-AUC** | **PR-AUC** | **ROC-AUC** | **PR-AUC** | **ROC-AUC** | **PR-AUC** |
| KSMoFinder | **0.828+0.006** | **0.81+0.007** | **0.899+0.005** | **0.746+0.013** | **0.851+0.008** | **0.839+0.008** | **0.914+0.003** | **0.79+0.009** |
| ProtT5 | 0.714**+**0.01 | 0.686**+**0.012 | 0.851**+**0.05 | 0.61**+**0.014 | 0.752**+**0.007 | 0.726**+**0.001 | 0.871**+**0.04 | 0.676**+**0.009 |
| ESM2 | 0.659**+**0.012 | 0.626**+**0.011 | 0.836**+**0.004 | 0.563**+**0.011 | 0.691**+**0.009 | 0.659**+**0.01 | 0.854**+**0.003 | 0.615**+**0.008 |
| ESM3 | 0.501**+**0.004 | 0.5**+**0.002 | 0.494**+**0.003 | 0.25**+**0.001 | 0.501**+**0.003 | 0.5**+**0.002 | 0.493**+**0.002 | 0.26**+**0.001 |

Table 3. Comparative evaluation results of KSMoFinder and Phosformer-ST (assessment 1) on testing datasets with and without easy test scenarios.

| **KSMoFinder vs Phosformer-ST (sub-assessment 1)** | | | | | | | | |
| --- | --- | --- | --- | --- | --- | --- | --- | --- |
| **Model** | **Without easy test scenarios** | | | | **With easy test scenarios** | | | |
|  | **Testing dataset 1** | | **Testing dataset 2** | | **Testing dataset 1** | | **Testing dataset 2** | |
|  | (Positive: 2276;  Negative: 2276) | | (Positive: 2276;  Negative: 7238) | | (Positive: 2735;  Negative: 2735) | | (Positive: 2735;  Negative: 8225) | |
|  | **ROC-AUC** | **PR-AUC** | **ROC-AUC** | **PR-AUC** | **ROC-AUC** | **PR-AUC** | **ROC-AUC** | **PR-AUC** |
| KSMoFinder | **0.924+0.0** | **0.924+0.001** | **0.924+0.0** | **0.819+0.001** | **0.935+0.0** | **0.937+0.0** | **0.931+0.0** | **0.84+0.001** |
| Phosformer-ST | 0.825+0.001 | 0.818+0.001 | 0.825+0.001 | 0.617+0.001 | 0.837+0.001 | 0.836+0.001 | 0.844+0.001 | 0.653+0.001 |
| **KSMoFinder vs Phosformer-ST (sub-assessment 2)** | | | | | | | | |
| **Model** | **Without easy test scenarios** | | | | **With easy test scenarios** | | | |
|  | **Testing dataset 1** | | **Testing dataset 2** | | **Testing dataset 1** | | **Testing dataset 2** | |
|  | (Positive: 281;  Negative: 281) | | (Positive: 281;  Negative: 7238) | | (Positive: 350;  Negative: 350) | | (Positive: 350;  Negative: 8225) | |
|  | **ROC-AUC** | **PR-AUC** | **ROC-AUC** | **PR-AUC** | **ROC-AUC** | **PR-AUC** | **ROC-AUC** | **PR-AUC** |
| KSMoFinder | **0.962+0.001** | **0.963+0.001** | **0.965+0.001** | **0.656+0.003** | **0.976+0.001** | **0.976+0.001** | **0.97+0.0** | **0.704+0.003** |
| Phosformer-ST | 0.96+0.001 | 0.941+0.002 | 0.949+0.0 | 0.358+0.003 | 0.964+0.001 | 0.948+0.002 | 0.953+0.0 | 0.389+0.003 |
| **KSMoFinder vs LinkPhinder** | | | | | | | | |
| **Model** | **Without easy test scenarios** | | | | **With easy test scenarios** | | | |
|  | **Testing dataset 1** | | **Testing dataset 2** | | **Testing dataset 1** | | **Testing dataset 2** | |
|  | (Positive: 2103;  Negative: 2103) | | (Positive: 2103;  Negative: 3203) | | (Positive: 2557;  Negative: 2557) | | (Positive: 2557;  Negative: 3901) | |
|  | **ROC-AUC** | **PR-AUC** | **ROC-AUC** | **PR-AUC** | **ROC-AUC** | **PR-AUC** | **ROC-AUC** | **PR-AUC** |
| KSMoFinder | **0.898+0.001** | **0.896+0.001** | **0.899+0.007** | **0.859+0.001** | **0.913+0.0** | **0.912+0.001** | **0.912+0.0** | **0.877+0.001** |
| LinkPhinder | 0.798+0.001 | 0.751+0.001 | 0.802+0.001 | 0.672+0.001 | 0.793+0.01 | 0.746+0.001 | 0.79+0.001 | 0.654+0.001 |
