# Supplementary material for: KSMoFinder—knowledge graph embedding of proteins and motifs for predicting kinases of human phosphosites
Source: Bioinform Adv. 2025 Nov 11;5(1):vbaf289. doi: 10.1093/bioadv/vbaf289 (PMC12664573; doi:10.1093/bioadv/vbaf289)
Supplement: vbaf289_Supplementary_Data [file vbaf289_supplementary_data.zip › Supplementary_File_3.docx]

This file contains the hyperparameters used to train the classifiers for different assessments.

The optimal hyperparameters of all the classifiers are determined using a 5-fold stratified cross-validation technique.

Table 1: Lists the final parameters used to train the classifiers for comparative evaluation of embeddings from the four KGE models (Assessment 1).

| **Embedding source** | **Neural Network - hidden layers size** | **Learning Rate** | **Epoch** |
| --- | --- | --- | --- |
| TransE | 40, 50 | 0.01 | 36 |
| DistMult | 40, 50 | 0.01 | 60 |
| ComplEx | 40, 50 | 0.001 | 42 |
| ExpressivE | 40, 50 | 0.001 | 11 |

Table 2: Lists the final parameters used to train the classifiers for comparative evaluation of embeddings from KSMoFinder-KGE and embeddings from external pre-trained models (Assessment 2).

| **Embedding source** | **Neural Network - hidden layers size** | **Learning Rate** | **Epoch** |
| --- | --- | --- | --- |
| KSMoFinder-KGE | 40, 50 | 0.001 | 73 |
| ESM2 | 90, 100 | 0.0001 | 63 |
| ESM3 | 80, 90 | 0.001 | 4 |
| ProtT5 | 80, 90 | 0.001 | 37 |
| Random | 40, 50 | 0.001 | 1 |

Table 3: Lists the final number of epochs used to train the classifiers for assessing the influence of additional feature embeddings from other pre-trained models (Assessment 3). All six classifiers are trained with two hidden layers of size, [40, 50], and a learning rate of 0.001.

| **Test No.** | **Test Description** | **Features & Embedding sources** | **Epoch** |
| --- | --- | --- | --- |
| 1 | With  KSMoFinder-KGE only | Protein biological associations, 9-mer motif (**KSMoFinder-KGE**) | 76 |
| 2 | Inclusion of ProstT5-based protein structure information | Protein biological associations , 9-mer motif (**KSMoFinder-KGE**)  Protein structure (ProstT5) | 36 |
| 3 | Inclusion of Phosformer-based kinase domain sequence and motif sequence information | Protein biological associations, 9-mer motif (**KSMoFinder-KGE**)  Kinase domain sequence, 15-mer motif (Phosformer) | 36 |
| 4 | Inclusion of ProstT5 (structure) and Phosformer (kinase domain sequence and motif sequence) information | Protein biological associations, 9-mer motif (**KSMoFinder-KGE**)  Kinase domain sequence, 15-mer motif (Phosformer)  Protein structure (ProstT5) | 44 |
| 5 | Effect of dropping KSMoFinder-KGE | Kinase domain sequence, 15-mer motif (Phosformer)  Protein structure (ProstT5) | 88 |
| 6 | Effect of dropping proteins’ biological information contributed via KSMoFinder-KGE | 9-mer motif (KSMoFinder-KGE)  Protein structure (ProstT5) | 27 |
